# A New Approach to the Determination of Biogenic Amines in Wine

**DOI:** 10.3390/molecules31010071

**Published:** 2025-12-24

**Authors:** Anna Salimova, Alexandra Vasilieva, Evgeniy Belyaev, Konstantin Sakharov, Sergey Andreev

**Affiliations:** 1 Disinfectology Institute of F.F. Erisman FSCH of Rospotrebnadzor, 18 Nauchny drive, 117246 Moscow, Russia; 2 School of Materials Science and Engineering, Nanyang Technological University (NTU), 50 Nanyang Avenue, Singapore 639798, Singapore

**Keywords:** biogenic amines, wine, precolumn derivatization, high-performance liquid chromatography, p-toluene sulfonyl chloride

## Abstract

Biogenic amines (BAs) can be found in various foods, such as cheese, wine, and chocolate. The consumption of a sufficient quantity of BA can lead to symptoms including headaches, hypertonia, and diarrhea. For this reason, the amount of BA in food is regulated in many countries. A new method for the determination of biogenic amines in wine has been proposed, which involves derivatizing BA with p-toluene sulfonyl chloride (TsCl) and using K_2_S_2_O_8_ to reduce the matrix effect. The derivatives of putrescine, cadaverine, histamine, and tyramine with TsCl were synthesized and characterized by ^1^H NMR spectroscopy. Separation of BA derivatives was performed using a reversed-phase high-performance liquid chromatography (RP-HPLC). The chromatographic system was equipped with a reversed-phase C8 column and a diode array detector. This method was validated to analyze the above-mentioned biogenic amines simultaneously in red and white wine samples. The detection limits for putrescine, cadaverine, histamine, and tyramine in wine samples were 0.0248 mg·L^−1^, 0.0645 mg·L^−1^, 0.346 mg·L^−1^ and 0.00866 mg·L^−1^, respectively. The calibration curves showed good linearity (r > 0.999), and biogenic amines recovery varied from 83.0 to 110%. The proposed method demonstrates high sensitivity, straightforward sample preparation, and rapid analysis time.

## 1. Introduction

Amines are a class of nitrogen-containing organic compounds that are of fundamental interest to chemists, biologists, and ecologists [1]. Their structural diversity, ranging from simple aliphatic to complex heterocyclic derivatives, gives rise to a wide variety of biological activities and practical applications. BA occupies a special place among amines. These low-molecular-weight compounds are formed by the decarboxylation of amino acids [2]. This process involves the removal of the α-carboxyl group, resulting in the formation of the corresponding amine. Virtually all amino acid decarboxylation reactions are catalyzed by decarboxylase enzymes [3,4]. Each BA is formed from a specific amino acid by a specific enzyme. For example, histamine is formed from the amino acid histidine by histidine decarboxylase. Lysine decarboxylase catalyzes the decarboxylation of lysine to form cadaverine, which can also be synthesized by ornithine decarboxylase under conditions of low ornithine and high lysine concentrations.

While many BAs play important roles in human physiology, some are considered by-products of metabolism. A particularly high degree of BA formation are found in foods such as cheese, wine, beer, chocolate, vegetables, fish products, and meat [5,6,7]. BA can accumulate in food during enzymatic processes, such as fermentation in the production of wine and beer, or during food spoilage in meat and fish. The amount of BA can reportedly be used as an indicator of product freshness and microbial contamination [8].

However, the consumption of high concentrations of BA can cause symptoms such as hives, hypotension, palpitations, diarrhea, headaches, nausea, and vomiting [6,9]. Putrescine and cadaverine can increase the toxicity of tyramine and histamine; additionally, they can react with nitrites to form carcinogenic N-nitroso compounds. In turn, putrescine can influence abnormal cell transformation [10].

Although excessive amounts of BAs in food can be dangerous, there are only a few legislative documents worldwide that control the BA content in food. For example, Commission Regulation (EC) No 2073/2005 of 15 November 2005 on microbiological criteria for foodstuffs sets out the maximum permitted level of histamine in fish products. Some EU countries have recommended standards for the content of histamine and putrescine in wine. In Germany and the Netherlands, the recommended level of biogenic amines in wine is up to 2 mg·L^−1^. France, Belgium, Switzerland and Australia recommend levels of up to 8–10 mg·L^−1^ [11]. Giglioti Research Group reported that the BA content in white wines is lower than in red wines, i.e., 0–10 mg·L^−1^ for white wines and 0–30 mg·L^−1^ for red wines [12].

Several analytical methods for determining BA have been described in the literature. These methods are based on separation using HPLC with UV/visible [13,14,15], fluorimetric [16,17] and MS/MS [18,19,20,21] detection. Preliminary derivatization is often used in the determination of BAs, since many BAs, due to their chemical structure, do not absorb the UV and do not contain chromophore groups. Preliminary derivatization most often involves the use of derivatives such as dansyl chloride, orthophthalaldehyde and benzoyl chloride [22]. The dansylation of BA is the most common method of derivatization [23] because of the high stability of the derivatives [24]. At the same time, dansylation often needs heating (up to 60 °C) or takes a long time.

The existing chromatographic methods for determining biogenic amines have several fundamental limitations. These include complex multistage sample preparation, insufficient sensitivity for detecting trace amounts of BA in complex food matrices; and the limited applicability of derivatization reagents, which are highly toxic and unstable in environments containing alcohol. Thus, this paper therefore proposes a new approach to solving these analytical problems, based on the use of a universal derivatization agent with improved toxicological and operational characteristics. Based on this approach, a method has been developed and validated for the simultaneous determination of the four priority biogenic amines (histamine, tyramine, putrescine, and cadaverine) in wines.

## 2. Results and Discussion

### 2.1. Synthesis and Characterization of BA Derivatives with TsCl

**Figure 1:**
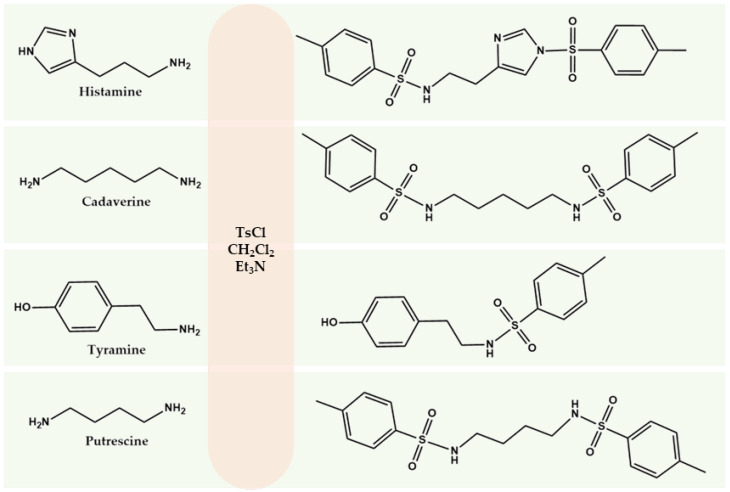
Scheme of BA derivatization with TsCl.

Here we propose the use of TsCl as a derivatization agent for determining BA in wine. Compared with the other reagents considered, TsCl provides high stability of derivatives, rapid reaction under mild conditions, and selectivity for amino groups in the presence of ethanol. The reaction mechanism is based on nucleophilic substitution, in which the sulfonyl group of TsCl interacts with the nitrogen atom of the amine to form a stable derivative containing an aromatic chromophore system. According to stoichiometry, one molecule of TsCl is required to replace one amino group (Figure 1).

To establish the optimal conditions, the reaction was carried out at various amine-to-tosyl chloride molar ratios (1:2, 1:3, 1:5 and 1:10). At the same time, the effect of stirring time on the yield of the derivative was investigated, with stirring times of 1, 3, 5, 10, 30, and 60 min being tested. The concentrations of TsCl and its derivatives were determined using UV/Vis spectrophotometry. Since histamine and tyramine have an absorbance in the same region as TsCl, this experiment was only performed for putrescine and cadaverine.

**Figure 2:**
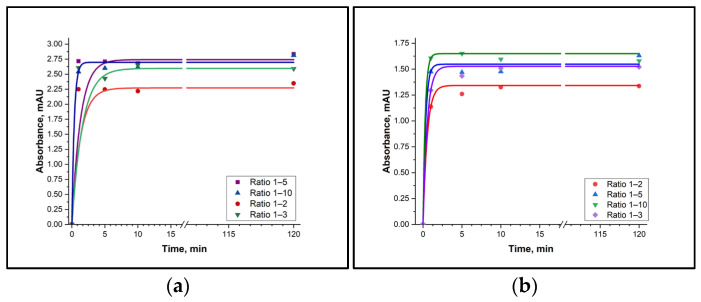
Time dependence of the BA derivative with TsCl formation. (**a**) Putrescine-TsCl derivative (absorbance measured at 227 nm); (**b**) Cadaverine-TsCl derivative (absorbance measured at 227 nm).

It was established that the derivatives of putrescine and cadaverine are formed in the solution with TsCl within 10 min, and that they remain stable when the reaction time is increased to 120 min (Figure 2). It should also be noted that the reaction was not fully complete when the BA:TsCl ratio was 1:1. Moreover, a fifth- and tenth-fold excess of TsCl did not lead to faster formation of the derivatives. Therefore, it can be concluded that the TsCl concentration must be at least twice as high as the BA concentration in real samples.

Although the biogenic amines considered in this study can be categorized into two structural groups—aliphatic diamines (putrescine and cadaverine) and aromatic amines (histamine and tyramine)—the results obtained for aliphatic amines, shown in Figure 2, were also valid for aromatic amines. When tyramine and histamine were mixed with TsCl in CH_2_Cl_2_ for 10 min, the yield of the derivative of each amine was above 95%. Then, the tyramine and histamine derivatives were separated using a thin-layer chromatography, dried and weighed, and their purity was established using HPLC.

The BA:TsCl ratio was set at 0.95:1 when standard samples were synthesized, eliminating the need for an additional purification step using thin-layer chromatography. In this case, the derivative was separated from the reagents by extraction in a chloroform-water system. The formation of the derivatives was confirmed by ^1^H NMR spectroscopy (Table 1). The derivatives of all the BAs under consideration, which were synthesized and isolated in this way with TsCl, were subsequently used for chromatographic method development and validation.

In the proton spectra of the putrescine and cadaverine product mixtures, characteristic signals of the putrescine group protons (Na, Nb and CH_3_) with integral intensities of 4, 4 and 6, respectively, were observed. This indicates that the reaction proceeds simultaneously at two positions of the nitrogen group (Table 1).

A study of the tosylation reaction of histamine and tyramine revealed peculiarities in their chemical behavior. In the case of tyramine, the tosylation reaction not only proceeded via the amino group, but also affects the hydroxyl group of the phenolic fragment. This leads to the formation of a mixture of products.

Histamine contains an imidazole ring with an attached aliphatic primary amino group and has different nucleophilic activities of functional groups. The aliphatic amino group is a strong nucleophile and reacts selectively with tosyl chloride first. The nitrogen atom of the imidazole ring subsequently reacts because of its lower nucleophilicity, forming a bond with the tosyl chloride molecule.

### 2.2. Optimization of Separation Parameters

Although existing methods have demonstrated the separation of BA derivatives mixtures using reversed-phase chromatography on columns with a C18 phase [25,26,27], it was not possible to reproduce the separation of the BA mixture under consideration in this study.

**Figure 3:**
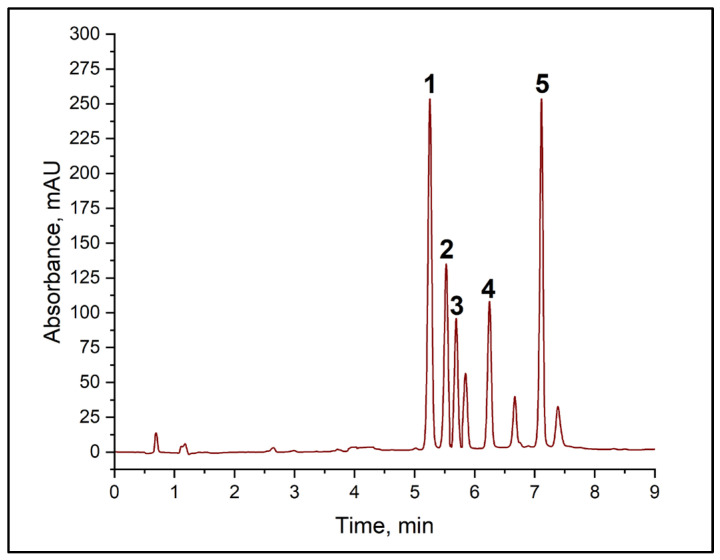
Chromatogram of a mixture of BA derivatives with TsCl. (1) putrescine derivative, (2) histamine derivative, (3) cadaverine derivative, (4) tosyl chloride, (5) tyramine derivative.

In the first stage, the potential for using normal-phase chromatography with NH_2_ phases in columns was explored. However, this approach did not enable the target analytes’ signals to be separated satisfactorily. The next stage involved the use of ion−pair chromatography with didecyl dimethyl ammonium chloride and alkylbenzenesulphonic acid as counterions to enhance the retention of amine derivatives. Although this approach offered improved selectivity compared with normal-phase chromatography, it could not achieve full resolution of all the peaks in the mixture under study. Subsequent optimization focused on reversed-phase chromatography using columns containing grafted phases of different structures: C18, C16, and C8. Results revealed that the combination of amine derivatives and the reagent (TsCl) was optimally separated using C8 and C18 columns. However, basic resolution (Rs < 1.5) between the histamine and cadaverine derivatives peaks was not achieved when using a C18 column, which is unacceptable for quantitative determination (see Table S1). The best results were obtained using the Acclaim C8 analytical column, which provided complete chromatographic resolution (Rs > 1.5) between all the target amine derivatives. A key advantage of the chosen system was the absence of interfering peaks from the wine matrix in the analyte retention region (BA). Figure 3 shows the typical chromatogram of the mixture of TsCl and BA derivatives, as well as the corresponding calibration curves, all of which have high correlation coefficients (see Table 2).

### 2.3. Determination of Biogenic Amines in Wine Samples

As mentioned previously, fermented products frequently contain high levels of biogenic amines. Wine is no exception, as evidenced by numerous studies. According to published data, the concentration of histamine in wine can reach 23.1 mg·L^−1^. Similar levels of histamine have been recorded in Italian wines (10.8 mg·L^−1^) and French wines (14.05 mg·L^−1^) [28]. Higher concentrations are also common for putrescine. In Italian wines, its concentration ranges from 11 to 31 mg·L^−1^ [29], whereas concentrations of up to 48 mg·L^−1^ have been reported in French samples [28]. These data confirm the importance of monitoring biogenic amines in wine products, as well as the need for reliable determination methods.

This work used samples of semisweet white and red wines to validate the methodology. When determining biogenic amines in wine, many matrix effects were identified that significantly complicate the analysis. The main interfering components are as follows:

- high ethanol content, which is the main product of fermentation;
- the presence of phenolic compounds, including phenolic acids and anthocyanins;
- the presence of other complex components, such as aldehydes, tannins and organic acids, which can affect derivatization and chromatographic separation processes.

The ‘spiked-in’ method was used to quantitatively assess the derivatization process, whereby BA standards were added to wine samples at various concentrations (2 and 20 mg·L^−1^ for each analyte).

Table 3 shows the results of different approaches to wine extraction using putrescine and cadaverine to minimize matrix effects. The main criterion for evaluating the efficiency of the sample preparation methods was the reaction yield, which should be between 80% and 120%. The results of the study show that extraction with organic solvents does not provide a satisfactory yield of the target analytes. This is due to the nature of biogenic amine derivatives, which have the ability to dissolve in both polar and nonpolar solvents.

Compared with extraction methods, the use of sorbents based on activated carbon, graphene, and carbon nanotubes significantly improved the reaction yield. Nevertheless, even with these materials, it was not possible to achieve the high yields required for determining target analytes.

As mentioned earlier, complex polyphenolic compounds interfere with the derivatization process. Furthermore, these compounds are present in significantly higher concentrations in red wine than in white wine. To address this issue, the oxidative destruction of aromatic structures using potassium peroxodisulphate (K_2_S_2_O_8_) was investigated as a potential solution. This method is based on generating sulphate radicals (SO_4_^−^•), which break down tannin macromolecules into low-molecular-weight fragments. This prevents the unwanted interactions between tosyl chloride and polyphenols, ensuring that they are consumed selectively in reactions with biogenic amines only.

To optimize the reaction, the influences of the concentration of K_2_S_2_O_8_ (0.01%, 0.1% and 1%) and the reaction time (5, 15, 30 and 60 min) were investigated. The most reproducible results were obtained using a 0.1% potassium peroxodisulphate solution mixed for 15 min. As shown in Table 4, the selected wine treatment method preserves the structures of the biogenic amines while reducing the matrix effect.

**Figure 4:**
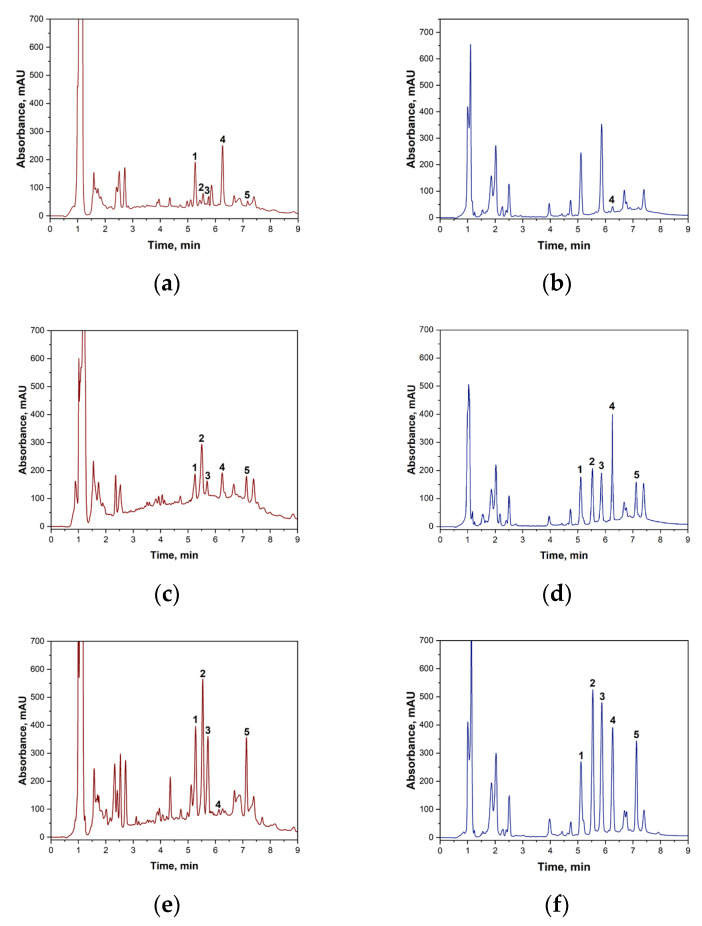
(**a**)—Blank sample of red wine with TsCl without BA addition; (**b**)—blank sample of white wine with TsCl without BA addition; (**c**)—red wine sample with the addition of a BA mixture (2 mg·L^−1^); (**d**)—white wine sample with the addition of a BA mixture (2 mg·L^−1^); (**e**)—red wine sample with the addition of a BA mixture (20 mg·L^−1^); (**f**)—white wine sample with the addition of a BA mixture (20 mg·L^−1^). 1—putrescine derivative signal; 2—histamine derivative signal; 3—cadaverine derivative signal; 4—tosyl chloride signal; 5—tyramine derivative signal.

Prior to the chromatographic determination of biogenic amines in wine using the ‘spiked-in’ method, the sample preparation procedure was validated. It was confirmed that potassium peroxodisulphate does not react with the target biogenic amines under the specified conditions. At the same time, blank wine samples were analyzed without the addition of standards to establish the background content of BA. The results showed that the white wine samples contained no amines within the method’s sensitivity range. The following concentrations of biogenic amines were found in red wine samples: putrescine: 2.13 mg·L^−1^; histamine: 2.17 mg·L^−1^; cadaverine: 1.31 mg·L^−1^; and tyramine: 0.877 mg·L^−1^. These background concentrations were taken into account in subsequent calculations of reaction yield and quantitative determination. Figure 4 shows the chromatograms of the original wine and the wine samples to which BA had been added.

The presented chromatograms demonstrate the effectiveness of the developed method. Complete chromatographic resolution (Rs > 1.5) is observed for all target analytes—histamine, tyramine, putrescine and cadaverine—as well as for unreacted tosyl chloride.

### 2.4. Method Validation Results

#### 2.4.1. Selectivity

The selectivity of the method was tested by analyzing red and white wine. Experiments revealed that the chromatographic peaks of the biogenic amine derivatives and tosyl chloride were separated. Therefore, the proposed method is suitable for identifying and quantifying putrescine, histamine, cadaverine, and tyramine in wine.

#### 2.4.2. The Limit of Detection (LOD) and Limit of Quantitation (LOQ)

The LOD and LOQ were assessed using the signal-to-noise ratio. According to the results presented in Table 4, the developed method demonstrated good sensitivity, as confirmed by the LOD values for each amine. The LOQ values were also calculated: 0.0752 mg L^−1^ for putrescine, 1.026 mg L^−1^ for histamine, 0.195 mg L^−1^ for cadaverine and 0.0269 mg L^−1^ for tyramine. The calculations were performed using the following signal-to-noise ratio criteria used to calculate LOD and LOQ: LOD = 3.3 × (σ/S) and LOQ = 10 × (σ/S), where σ is the standard deviation of the blank sample signal and S is the average slope of the calibration curve.

#### 2.4.3. Linearity

The calibration curves for each BA are linear (see Table 4), and in all the cases the correlation coefficient value exceeded 0.998. The correlation and slope coefficients, which were calculated using the least-squares deviation method for hydrogen peroxide, are shown in Table 4. The average correlation coefficient value exceeds 0.999, confirming that the developed method meets the set criteria.

#### 2.4.4. Precision and Accuracy

The precision and accuracy for six various concentrations of biogenic amines are included in Table 4. The R.S.D. (%) values that were used to assess the precision were within the range of 0.21–1.06% for hydrogen peroxide. The recovery coefficient that was used to assess the precision of the study varied within the range of 80.0–120.0% for hydrogen peroxide. Thus, the method developed can be combined with precision and accuracy.

A key advantage of the developed method is the absence of a wine matrix effect. In the region of the chromatogram where amine derivatives are eluted, no interfering peaks from wine components are observed. The method was validated in accordance with [30]. It demonstrated good linearity across the entire studied range, as evidenced by R^2^ values ranging from 0.9997 to 0.9998 for all analytes (see Table 4).

A comparative analysis (see Table 5) shows that the developed method is consistent and procedural. In some respects, it is also superior to existing methods for determining biogenic amines in wine in terms of sensitivity. The achieved limits of detection (LODs) are comparable to those of HPLC-MS-MS methods, although the proposed method does not require expensive mass spectrometry equipment. The main advantages of the proposed method are simplified sample preparation and the availability of the necessary equipment, making it possible to develop a method that can be used in modern routine analytical laboratories

## 3. Materials and Methods

### 3.1. Chemicals and Wine Samples

Standard samples of the following biogenic amines were purchased from Supelco: cadaverine (CAS No. 462-94-2), putrescine (CAS No. 110-60-1), tyramine (CAS No. 51-67-2) and histamine (CAS No. 51-45-6). Tosyl chloride (CAS No. 51-67-2), triethylamine (CAS No. 121-44-8), and potassium peroxodisulfate (CAS No. 7727-21-1) were purchased from Merck (Darmstadt, Germany). The reagents and solvents required for NMR and HPLC analysis were also purchased from Merck (Darmstadt, Germany) and Scharlau (Barcelona, España). Chloroform (CAS No. 67-66-3), ethyl acetate (CAS No. 141-78-6), heptane (CAS No. 142-82-5), methanol (CAS No. 67-56-1), acetonitrile (CAS No. 75-05-8) of HPLC grade were also purchased from Merck (Darmstadt, Germany).

Wine samples were bought in the nearest supermarket.

### 3.2. HPLC Instrumentation and Chromatographic Conditions

The HPLC system used was a Thermo ULTIMATE 3000 (Thermo Fischer Scientific, Waltham, MA, USA) instrument equipped with a DAD-3000 diode array detector (Thermo Fischer Scientific). This device is also supplied with a column thermostat, an autosampler with a 20 μL loop and a gradient pump with mixing on the low pressure side for obtaining a 4-component gradient with a built-in degassing device.

In this study the following analytical columns were used: Luna NH_2_, 5 µm, 100 Å, 150 × 4.6 mm and Luna CN Luna, 5 µm, 100 Å, 250 × 4.6 mm (supplied by Phenomenex, Torrance, CA, USA); Acclaim C18, 120 Å, 5 µm, 150 × 4.6 mm, Acclaim C16, 120 Å, 3 µm, 150 × 2.1 mm, Acclaim C8, 120 Å, 5 µm, 250 × 4.6 mm (all supplied by Thermo Fischer Scientific, Waltham, MA, USA).

The best separation was achieved using an Acclaim C8 column (150 × 4.6 mm, 5 µm) and a mobile phase consisting of water (phase A) and acetonitrile (phase B). The gradient program used was as follows: 0–1 min: 40.0% B; 6.0–10.0 min: 70.0% B; 12.0–15.0 min: 7.0% B. The column temperature was set to 38 °C, the injection volume was 8 µL per injection and the liquid flow rate was 0.8 mL/min. UV detection was performed at wavelengths between 220 and 250 nm. The total analysis run time was 15 min.

The BA stock solution with a concentration of 400 mg·L^−1^ was prepared by dissolving an accurately weighed amount of the standard sample in a 25 mL volumetric flask containing acetonitrile.

### 3.3. Wine Sample Preparation

The wine samples were prepared according to the following procedure: a 10 mL volumetric flask was filled with 1.0 mL of a 0.1% solution of potassium peroxodisulphate (K_2_S_2_O_8_) and an amine solution, and its volume varied from 0.2 to 1.0 mL. The total volume was made up of the wine (red or white). The mixture was stirred for 10 min. After that 3.0 mL of the intermediate solution was transferred to a 10 mL volumetric flask and 5.0 mL of a 6000 mg·L^−1^ tosyl chloride solution and 0.083 mL of triethylamine were added.

The tosyl chloride solution was prepared by dissolving a precise weight of the reagent in acetonitrile, after which the solution was brought up to the mark in a 25 mL volumetric flask. This concentration was optimised experimentally to ensure the complete derivatisation of the target amines while maintaining an excess of reagent.

The total volume was made up of acetonitrile, then the mixture was stirred for 15 min more. Prior to chromatographic analysis, the sample was filtered through a 0.45 μm membrane filter and transferred to a chromatographic vial.

### 3.4. NMR

^1^H NMR spectra were recorded on a Bruker Avance III spectrometer operating at 600 MHz in a CDCl_3_ solution at room temperature. Chemical shifts are given relative to the residual proton signal of the deuterated solvent (δ = 7.26 m.d. for CDCl_3_).

### 3.5. Data Analysis

Chromatographic data were collected and processed via Chromeleon 7 software (Thermo Fischer Scientific, Waltham, MA, USA). Excel 2019 (Microsoft Corporation, Redmond, WA, USA) and OriginPro 2021 (Origin Corp., Northampton, MA, USA) were used for detailed calculations and plotting.

## 4. Conclusions

The method developed and validated in this research proved its high efficiency for determining biogenic amines in wine, including preliminary derivatization with TsCl, followed by HPLC-DAD analysis. The use of TsCl as a universal derivatization agent for the simultaneous determination of primary and secondary amines was proposed for the first time, providing significant advantages over existing analogues in terms of derivative stability and selectivity in alcohol-containing media.

According to the data in Table 4, there are numerous chromatographic methods for determining biogenic amines in wine. An analysis of the literature has revealed several limitations of the existing approaches. The use of a mass-spectrometric detection for the determination of amines does not provide advantages in terms of sensitivity. A significant drawback of many published articles is their labor input and time-consuming processes, which are associated with multistage sample preparation. This includes prolonged mixing (up to 120 min) and solid-phase extraction procedures.

In contrast to the methods reviewed, the approach developed in this study has the following advantages:

- Facilitated sample preparation procedure
- Reduced analysis time
- Sensitivity corresponding to the real concentration ranges found in wine.
- Retention of high selectivity and accuracy of determination.

The proposed method provides optimal performance characteristics for a routine monitoring of biogenic amines in wineries.

A new approach to eliminate of matrix effects using potassium peroxodisulphate was developed to ensure the destruction of polyphenolic compounds in wine while preserving the integrity of the target analytes. This sample preparation method has been shown to be more efficient, fast and selective than all known alternatives. The practical application of the method for quality control and harmlessness of wine products has been demonstrated by testing it on real wine samples.

## Figures and Tables

**Table 1:** Selected signals (in ppm) of the 1H-NMR spectra of TsCl and BA derivatives.

Compound	Tosyl Benzene Ring	NH	Putrescine CH_2_	Cadaverine CH_2_	Tyramine Benzene Ring	Tyramine CH_2_	Histamine Imidazole Ring	Histamine CH_2_
Tosyl chloride	2.50							
	7.55							
	8.00							
Putrescine derivative	2.44	5.58	1.4					
	7.39		1.97					
	8.71							
Cadaverine derivative	2.50	5.59		1.2				
	7.55			1.34				
	8.00			2.76				
Tyramine derivative	2.45	5.56			7.11	2.63		
	7.40				7.43	2.71		
	7.70							
Histamine derivative	2.43	5.78					7.39	3.07
	7.46						7.70	3.20
	7.88							

**Table 2:** BA derivatives’ chromatogram parameters.

BA Derivative	Retention Time	Peak Asymmetry	Match *	Resolution	R^2^
Putrescine	5.25	0.99	998	2.32	0.9995
Histamine	5.53	0.97	999	1.50	0.9997
Cadaverine	5.69	0.98	999	1.51	0.9995
Tyramine	7.11	1.0	1000	1.52	0.9991

* Match (the match factor) is a dimensionless quantity.

**Table 3:** Optimization of BA extraction.

Pretreatment	Extracting Agent	Wine	Recovery, %	
			Putrescine	Cadaverine
Liquid–liquid extraction	Chloroform	White	72.2	99.8
		Red	31.5	41.5
Liquid-liquid extraction	Ethyl acetate	White	45.8	43.6
		Red	42.6	37.5
Liquid-liquid extraction	Heptane	White	74.1	37.5
		Red	50.9	29.3
Solid-phase extraction	Activated carbon	White	55.7	80.1
		Red	28.9	13.3
Solid-phase extraction	Graphene	White	59.0	89.9
		Red	53.9	78.1
Solid-phase extraction	MWCNT	White	52.6	102.8
		Red	44.0	101.3

**Table 4:** Method validation results.

Parameter	Putrescine	Histamine	Cadaverine	Tyramine	Criteria
Retention time, min	5.267	5.533	5.700	7.113	
Repeatibility—retention time R.S.D (%)	0.08	0.05	0.05	0.03	<1%
Repeatibility—area R.S.D (%)	0.86	0.77	0.39	0.46	<1%
Theoretical plates	31,265	32,259	35,350	78,843	>2000
Theoretical plates R.S.D (%)	3.91	3.68	2.94	6.32	
Resolution	2.27	3.11	2.65	2.31	>1.5
Resolution R.S.D (%)	1.59	2.35	1.80	16.35	
Asymmetry	0.86	0.90	0.84	0.92	<2
Asymmetry R.S.D (%)	0.80	3.00	6.32	3.11	
Linearity range, mg·L^−1^	1.50–37.0	1.50–21.5	2.5–30.5	1.25–20.0	
Correlation coefficient	0.9994	0.9998	0.9999	0.9990	>0.9990
Intercept	−1.00 ± 0.09	−0.824 ± 0.057	−0.829 ± 0.113	−0.92 ± 0.06	
Slope	1.38 ± 0.01	1.05 ± 0.01	1.73 ± 0.01	1.25 ± 0.11	
LOD (mg L^−1^)	0.0248	0.0645	0.346	0.00886	
LOQ (mg L^−1^)	0.0752	0.195	1.026	0.0269	
Recovery in red wine, %	86.8	85.6	90.7	103	80–120
Recovery in white wine, %	88.9	80,9	104	110	80–120

**Table 5:** Method validation results.

Quantitation	Detection	Biogenic Amine	Derivatization	Pretreatment	Detection Limits, µg·L^−1^	References
HPLC	FL	Putrescine, cadaverine, histamine, tyramine	OPA	Automated pre-column	33	[31]
HPLC	FL	Putrescine, cadaverine, histamine	DNS-Cl	Salting-out-assisted LLE	28 7 220	[24]
UPLC	Q-TOF-MS	Putrescine, cadaverine, histamine	DNS-Cl	SPE	5 10 10	[32]
GC	MS	Putrescine, cadaverine, histamine, tyramine	ICBF	SPE	1 1 1 1	[33]
GC	MS	Putrescine, cadaverine, histamine, tyramine	Isobutyl chloroformate	DI-SPME	0.025 0.125 0.155 0.009	[34]
HPLC	MS/MS	Putrescine, cadaverine, histamine, tyramine	TsCl		7.3 10 3.8 11	[35]
HPLC	DAD	Histamine, tyramine	CNBF	-	320 360	[36]
Capillary electrophoresis	MS/MS	Putrescine, cadaverine, histamine, tyramine		PVPP	2 2 2 2	[37]
HPLC	DAD	Putrescine, cadaverine, histamine, tyramine	TsCl	K_2_S_2_O_8_	24.8 64.5 346 8.86	Present work

## Data Availability

Data are contained within the article and Supplementary Materials.

## Supplementary Materials

The following supporting information can be downloaded at: https://www.mdpi.com/article/10.3390/molecules31010071/s1, Table S1: Selected chromatograms of a mixture of putrescine, histamine, cadaverine and tyramine derivatives with TsCl.

## Abbreviations

The following abbreviations are used in this manuscript:

BA	Biogenic amines
HPLC	High-performance liquid chromatography
TsCl	p-Toluene sulfonyl chloride (Tosyl chloride)
MWCNT	Multiwall carbon nanotube
UV	Ultraviolet region of spectrum
MS/MS	Tandem mass spectrometry
OPA	o-Phthaldialdehyde
Dns-Cl	5-(Dimethylamino)naphthalene-1-sulfonyl chloride (Dansyl chloride)
CNBF	1-Chloro-2,4-dinitro-5-(trifluoromethyl)benzene
PVPP	Polyvinylpolypyrrolidone
