# Supplementary material for: A New Approach to the Determination of Biogenic Amines in Wine
Source: Molecules. 2025 Dec 24;31(1):71. doi: 10.3390/molecules31010071 (PMC12786502; doi:10.3390/molecules31010071)
Supplement: Supplementary file 1 [file molecules-31-00071-s001.zip › molecules-4010880-supplementary.pdf]

Table S1. Selected chromatograms of a mixture of putrescine, histamine, cadaverine and tyramine derivatives with TsCl

All chromatograms were detected at the wavelength 220 nm.

NH2 4.6x150 mm

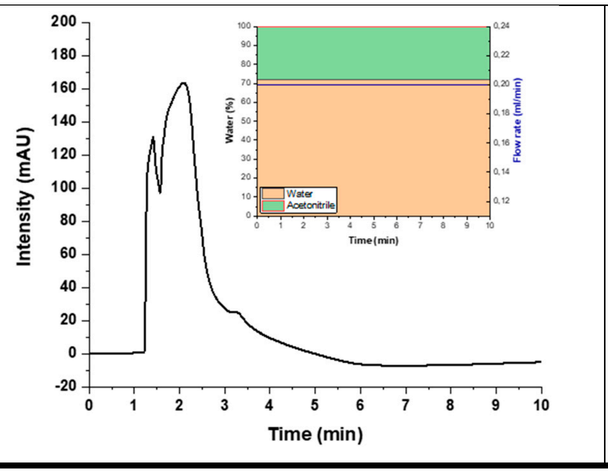

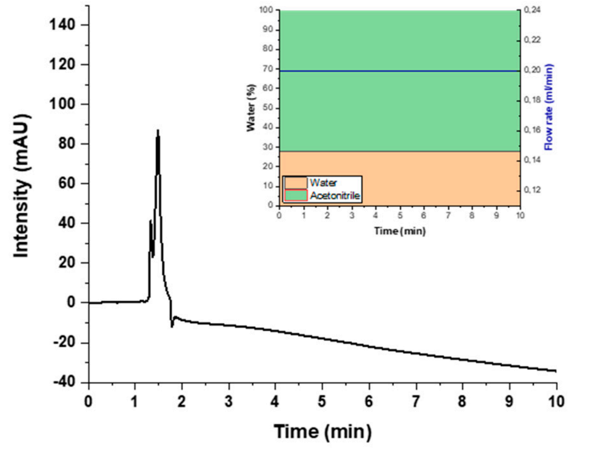

C 18 Zorbax 4.6x150 mm ion-pair chromatography using LABS

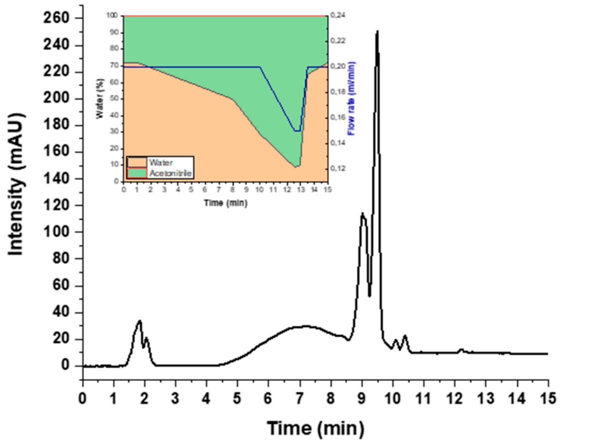

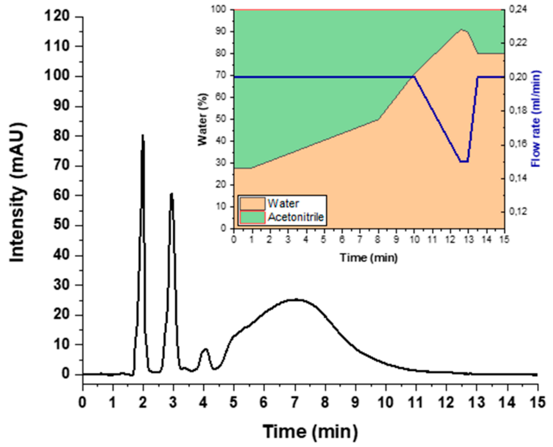

C 18 Zorbax 4.6x150 mm ion-pair chromatography using DDAC

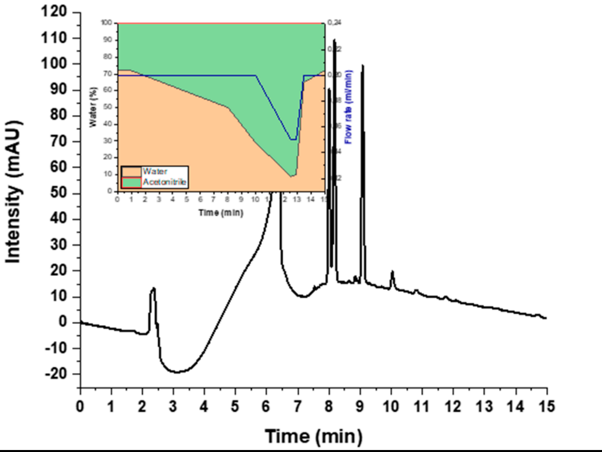

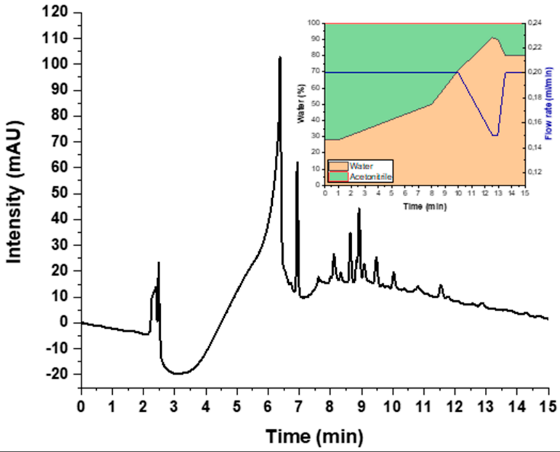

C 16 Acclaim 2.1x150 mm reverse-phase chromatography

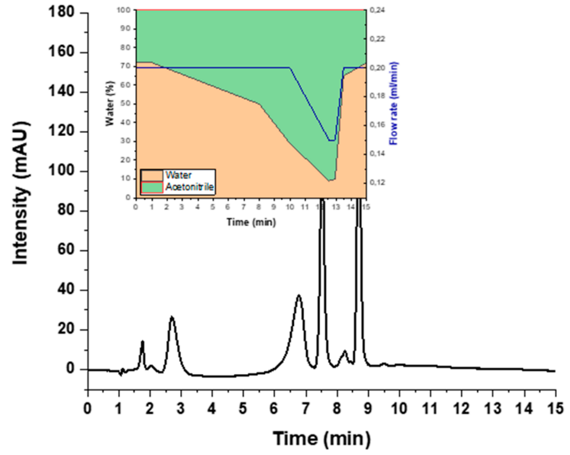

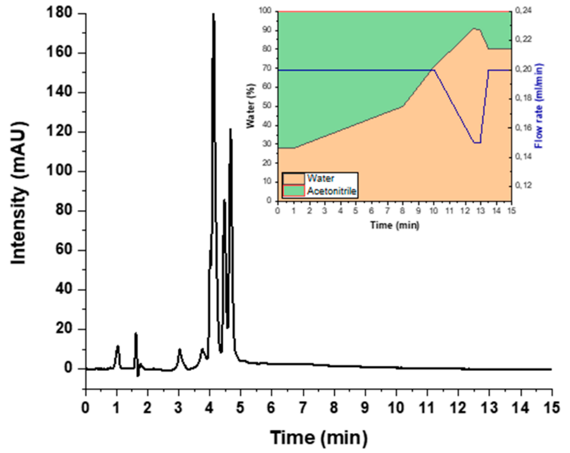

C 18 Zorbax 4.6x150 mm reverse-phase chromatography

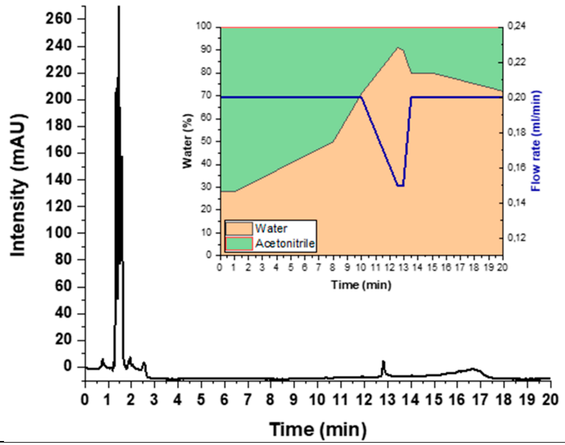

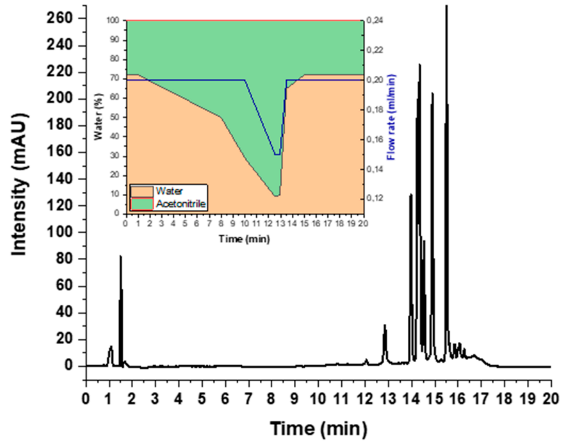

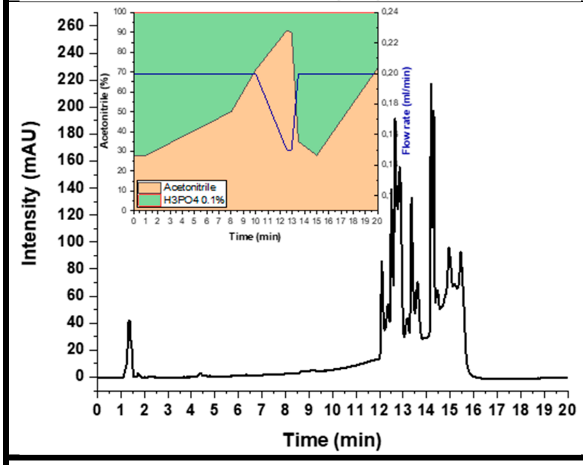

C 18 Acclaim 2.1x150 mm reverse-phase chromatography

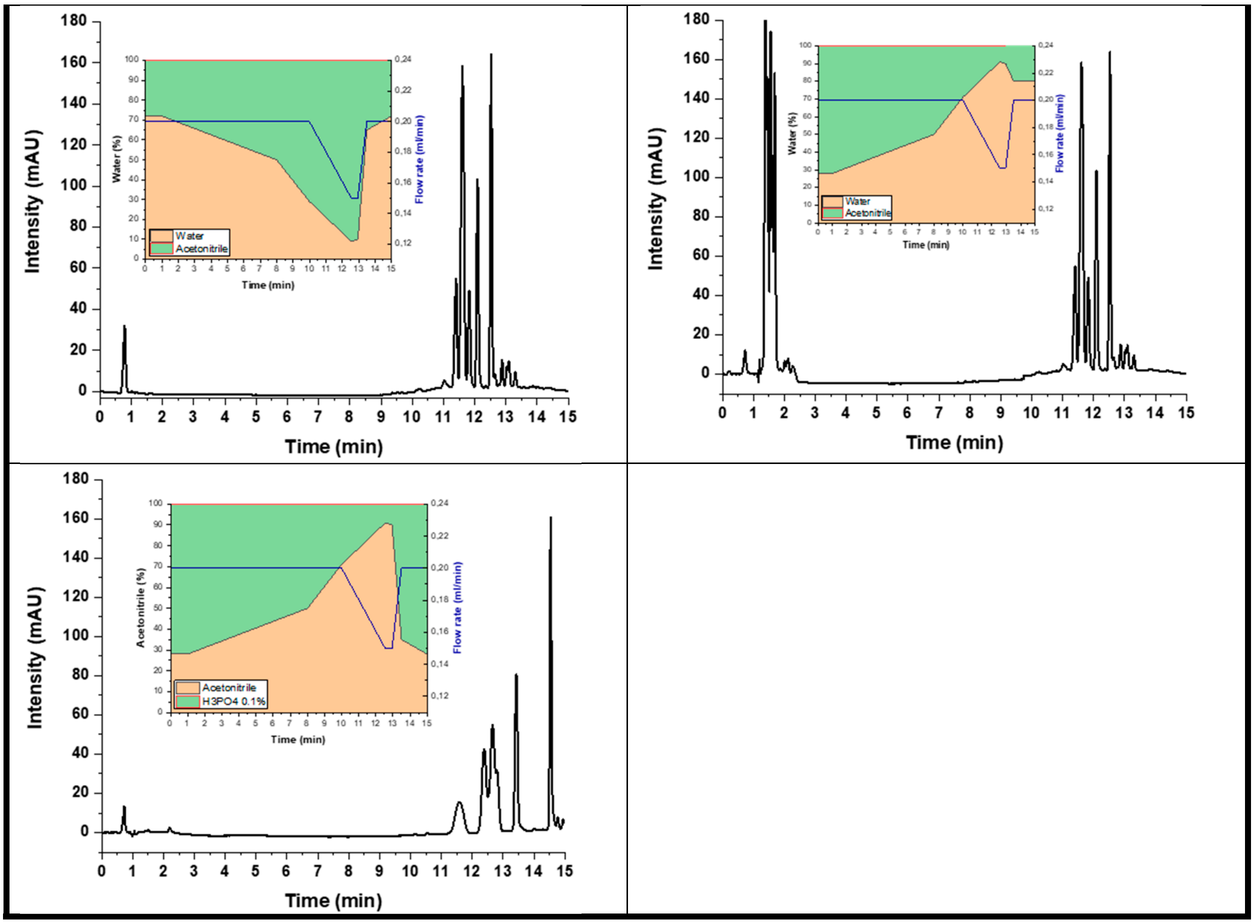

Intensity (mAU)
Time (min)
Water (%)
Flow rate (ml/min)
Water
Acetonitrile
Acetonitrile (%)
H3PO4 0.1%
